# CLEC3B as a Potential Prognostic Biomarker in Hepatocellular Carcinoma

**DOI:** 10.3389/fmolb.2020.614034

**Published:** 2021-01-20

**Authors:** Xing-Wei Xie, Shan-Shan Jiang, Xiang Li

**Affiliations:** ^1^College of Plant Protection, Henan Agricultural University, Zhengzhou, China; ^2^Key Laboratory of Forensic Toxicology of Herbal Medicines, Guizhou Education Department, School of Basic Medicine, Guizhou University of Traditional Chinese Medicine, Guiyang, China

**Keywords:** CLEC3B, hepatocellular carcinoma, prognosis, biomarker, tumor immunity

## Abstract

C-Type Lectin Domain Family 3 Member B (CLEC3B) encodes proteins associated with tumor invasion and metastasis. However, the interrelation between *CLEC3B* gene expression, tumor immunity, and prognosis of patients with hepatocellular carcinoma (HCC) is unclear. This study was conducted to investigate the prognostic potential of *CLEC3B* and its association with tumor tissue infiltration markers. *CLEC3B* expression was examined using the TIMER and Oncomine databases, with its prognostic potential assessed using the GEPIA and Kaplan–Meier plotter databases. The relationship between *CLEC3B* and tumor immune cell infiltration biomarkers was analyzed using TIMER. Here, we revealed that *CLEC3B* expression was decreased in HCC and was correlated with a poor survival rate in patients with HCC. Additionally, the expression of *CLEC3B* was negatively correlated with differential immune cell infiltration and various immune biomarkers. These results indicate a potential mechanism by which the expression of *CLEC3B* might adjust tumor immunity by modulating the infiltration of HCC immune cells. Our study demonstrated that *CLEC3B* could be a potential prognostic biomarker and might be involved in tumor immune cell infiltration in HCC.

## Introduction

Hepatocellular carcinoma (HCC) remains to be the second most fatal cancer worldwide, with an estimated incidence rate exceeding 1 million cases per year (Bray et al., 2018; Llovet et al., 2018). The major risk factors for HCC include non-alcoholic fatty liver disease, alcoholic hepatitis, and hepatitis (chronic hepatitis B and chronic hepatitis C) (Zhang et al., 2004; Makarova-Rusher et al., 2016). HCC incidence and mortality have decreased recently due to improved early diagnostic and surgical treatment techniques. However, recurrence and metastasis rates remain high, with a 5-year survival rate of only 20–30% (Pang et al., 2008; Yang et al., 2009). Additionally, some patients are not eligible for curative treatment due to late diagnosis (Huang et al., 2010). Thus, there is an urgent need for a deeper understanding of the molecular mechanism underlying HCC, which may lead to the development of novel therapeutic methods to increase long-term survival.

C-Type Lectin Domain Family 3 Member B (CLEC3B), a member of the C-type lectin superfamily that encodes tetragonal proteins in cells (Tanisawa et al., 2017; Liu et al., 2018), is a transmembrane Ca^2+^-binding protein, which is located in the cell plasma, extracellular matrix, and exosomes. CLEC3B has been reported to function in the mineralization process in osteogenesis and neuroprotection (Iba et al., 2013; Chen et al., 2015). Additionally, it is associated with tumor invasion and metastasis via plasminogen activation, leading to extracellular proteolysis (Clemmensen et al., 1986; Obrist et al., 2004; Danø et al., 2005; Dai et al., 2019). Furthermore, CLEC3B has been identified in various oncological pathologies, including breast, bladder, cervical, and ovarian cancers, melanoma, and gastric adenocarcinoma (Verspaget et al., 1994; Arvanitis et al., 2002; Brunner et al., 2007; Looi et al., 2009; Zhang et al., 2017). Although CLEC3B levels have been reported to decrease in patients with HCC, its specific role and associated tumorigenesis mechanisms remain unclear.

This study was conducted to investigate the prognostic potential of CLEC3B and its association with tumor tissue infiltration markers. *CLEC3B* expression was examined using the TIMER and Oncomine databases, with its prognostic potential assessed using the GEPIA and Kaplan–Meier plotter databases. The relationship between *CLEC3B* tumor immune cell infiltration biomarkers was analyzed using TIMER. Our study demonstrated that *CLEC3B* may play an important role in the prognosis of HCC. The results indicated a potential mechanism by which the *CLEC3B* expression might adjust tumor immunity by modulating the infiltration of immune cells in HCC patients.

## Materials and Methods

### Oncomine Database Analysis

Oncomine (https://www.oncomine.org/resource/login.html) is currently the world's largest cancer related-gene microarray database and integrated data-mining platform. Oncomine collects the most complete spectra of cancer mutations, related gene expression profiles, and relevant clinical information that can be used to discover new biomarkers or therapeutic targets. In the current study, the *CLEC3B* gene was selected as our research object and its expression levels in cancer and normal tissues were compared. The expression levels were considered significantly different in different tissues when fold change > 1.5, with *P* value > 0.001. We set the threshold value of gene rank to “top 10%” and the data type to “all” (Rhodes et al., 2007).

### Kaplan–Meier Plotter Database Analysis

By utilizing the RNA-seq data in the TCGA, EGA, and GEO databases, the Kaplan–Meier plotter (http://kmplot.com/analysis/) can evaluate the effect of more than 54,000 biomolecules on the survival rate in various tumor tissues. Here, the Kaplan–Meier plotter was used to analyze the association between *CLEC3B* expression and the survival rate for patients with liver, breast, lung, ovarian, and gastric cancers, which was determined based on the hazard ratios (HRs) and log-rank *P* values (Lánczky et al., 2016). We searched the database using *CLEC3B* as the input and carried out analysis with all the samples in the database with the following parameters: group cutoff: median; hazards ratio: yes; 95% confidence interval: yes.

### TIMER Database Analysis

Using TIMER 2.0 (http://timer.comp-genomics.org/), we analyzed the infiltration levels of immune cells in 31 tumor types, and more than 10,000 samples were extracted from the TCGA database. The TIMER database was used to determine the abundance of tumor infiltrates based on gene expression analysis (Li et al., 2017). The gene name *CLEC3B* under the DiffExp module with default parameters was used for obtaining the different expression levels in normal or tumor tissues. We investigated the relationship between *CLEC3B* expression and immune cell infiltration levels based on biomarker gene expression in various tumors under the gene module. Here, we chose *CLEC3B* as input and in turn detected cancer cells. B cells, CD8+ T cells, CD4+ T cells, neutrophils, macrophages, and dendritic cells were selected as the test types according to Li et al. (2016; Danaher et al., 2017). We further analyzed the expression correlation between *CLEC3B* and the biomarker genes of tumor-infiltrating immune cells, such as B cells, tumor-associated macrophages (TAMs), M1 macrophages, M2 macrophages, neutrophils, and dendritic cells (Sousa and Määttä, 2016; Danaher et al., 2017). Gene expression values were transformed to log2 RSEM values.

### GEPIA Database Analysis

GEPIA (http://gepia.cancer-pku.cn/index.html) is a publicly accessible online database and is used to analyze the expression of RNA sequencing data based on the GTEx and TCGA databases (Tang et al., 2017), with 9,736 and 8,587 tumor samples, respectively. We generated survival curves for disease-free survival and overall survival based on GEPIA using the log-rank test and the Mantel-Cox test. GEPIA was also used to analyze the gene expression correlation in various tumors with the given TCGA expression dataset. The correlation coefficient was determined by the Spearman method with default parameters.

## Results

### Expression of CLEC3B in HCC and Other Cancers

**Figure 1:**
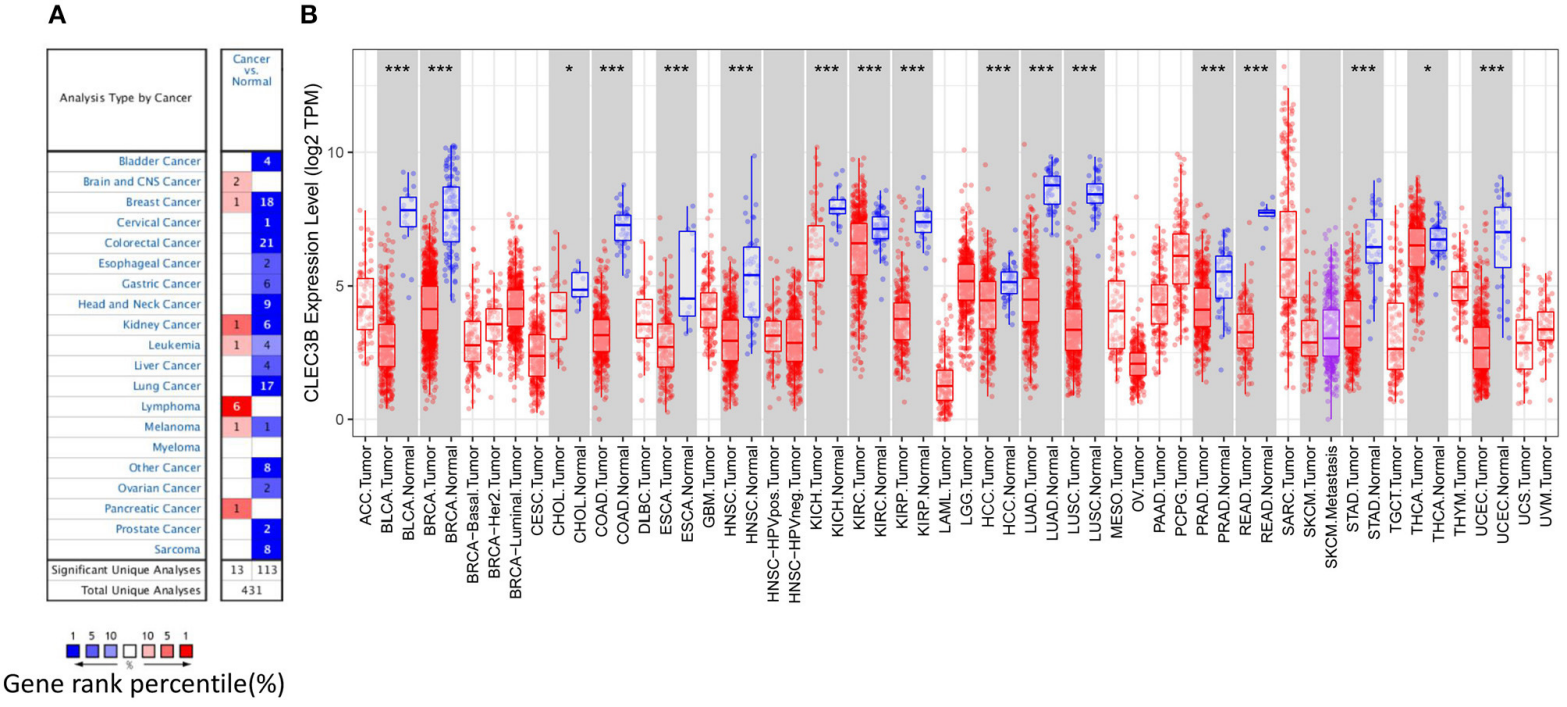
*CLEC3B* expression levels in various types of cancer tissues or tumor cells. **(A)** Expression levels of *CLEC3B* in various normal or cancer tissues via Oncomine analysis. **(B)** Expression levels of *CLEC3B* in various normal or tumor cells via TIMER analysis (*P < 0.05, ***P < 0.001).

To investigate the role of CLEC3B in carcinogenesis, we first measured the transcription levels of *CLEC3B* mRNA in various types of cancer tissues by Oncomine analysis. Except for lymphoma, brain and central nervous system cancer, and pancreatic cancer, the expression levels of the *CLEC3B* gene were significantly downregulated in all other available cancer tissues compared with normal tissues, including liver, bladder, breast, colorectal, cervical, esophageal, gastric, head and neck, lung, ovarian, prostate, and sarcoma tissues (Figure 1A). Similarly, RNA-seq data collected from the TCGA database indicated that low expression of *CLEC3B* was detected in 17 types of cancers, including HCC (Figure 1B). These findings strongly suggest that *CLEC3B* was inhibited and might play a negative role in the development of cancers.

### Prognostic Potential of CLEC3B Expression in HCC

The Kaplan–Meier plotter was used to assess the prognostic value of *CLEC3B* transcriptional changes in tumor tissues. We found that the low expression of *CLEC3B* in tumor tissues was correlated with poor prognosis, for example, poor first progression survival, overall survival, and post-progression survival in lung cancer; poor post-progression survival and overall survival in ovarian cancer; poor post-progression survival in gastric cancer; and poor relapse-free survival in breast cancer (Supplementary Figure 1). Additionally, the effect of *CLEC3B* expression on the prognostic potential of different tumors was analyzed via TCGA RNA-seq data in the GEPIA database. The low expression of *CLEC3B* was correlated with poor overall survival in HCC, head and neck squamous cell carcinoma, kidney renal clear cell carcinoma (KIRC), lower grade glioma (LGG), lung adenocarcinoma (LUAD), poor disease-free survival in HCC, esophageal carcinoma, KIRC, LGG, pancreatic adenocarcinoma, and thyroid carcinoma (Supplementary Figure 2).

**Figure 2:**
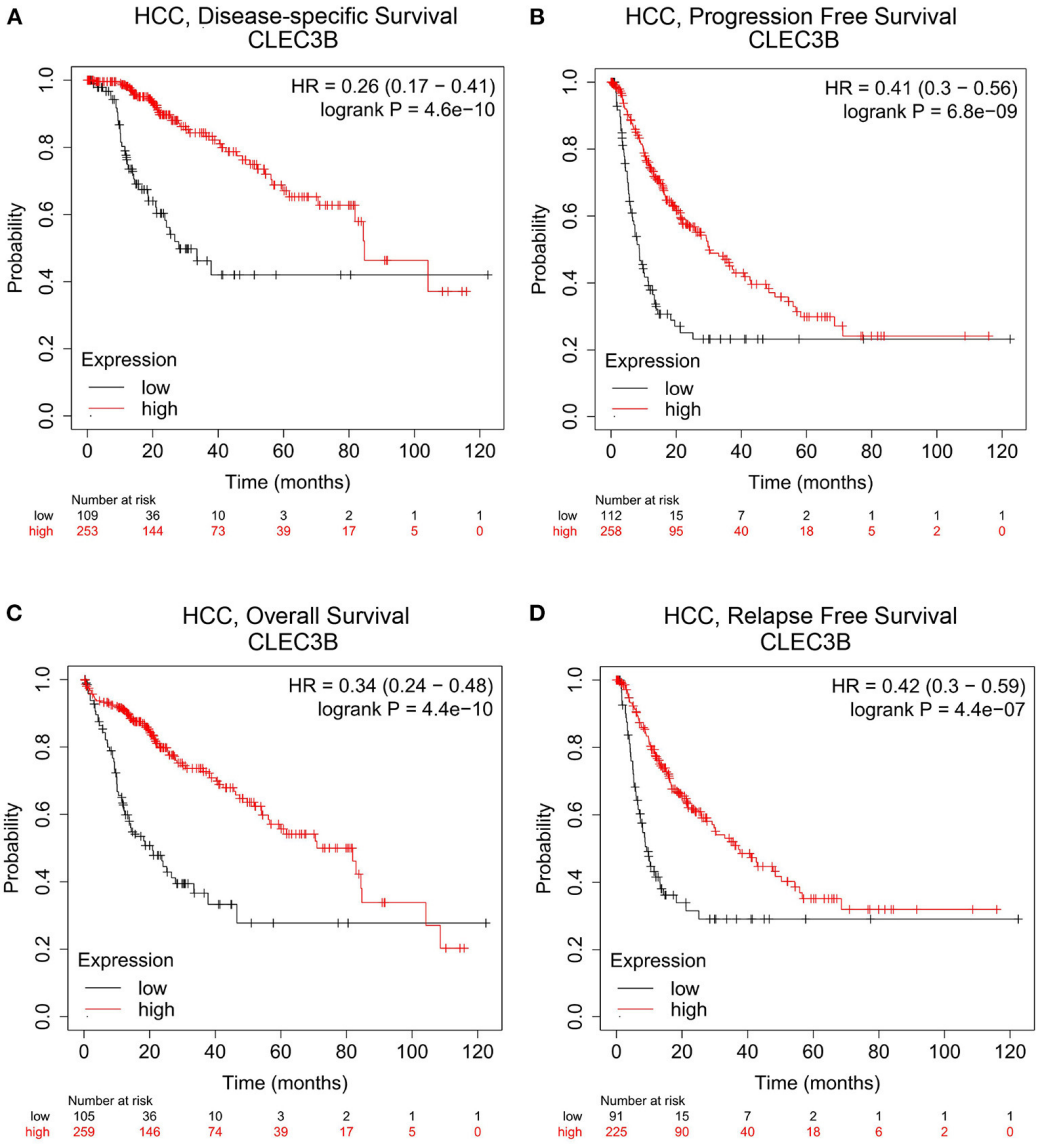
Correlation analysis between *CLEC3B* expression and prognostic survival in HCC patients via Kaplan–Meier plotter analysis. **(A)** Disease-specific survival, *n* = 357; **(B)** progression-free survival, *n* = 366; **(C)** overall survival, *n* = 364; **(D)** relapse-free survival, *n* = 313.

In particular, for HCC, we found that the disease-specific survival (Figure 2A, HR = 0.26, 95% CI = 0.17 to 0.41, *P* = 4.6e−10), progression-free survival (Figure 2B, HR = 0.41, 95% CI = 0.3 to 0.56, *P* = 6.8e−09), overall survival (Figure 2C, HR = 0.34, 95% CI = 0.24 to 0.48, *P* = 4.4e−10), and relapse-free survival (Figure 2D, HR = 0.42, 95% CI = 0.3 to 0.59, *P* = 4.4e−07) were significantly reduced when the expression level of *CLEC3B* was low.

We further analyzed the correlation between *CLEC3B* expression and various clinical features in HCC using the Kaplan–Meier plotter (Table 1). The low expression of *CLEC3B* was associated with poor progression-free survival and overall survival regardless of gender, race, stage or grade of tumor, alcohol consumption, or other status, suggesting that low expression of *CLEC3B* may be one of the causes of poor prognosis, and this gene could be an prognostic biomarker for HCC patients.

**Table 1:** Correlation of *CLEC3B* expression and prognosis in hepatocellular carcinoma patients with different clinicopathological factors via Kaplan–Meier plotter.

**Clinicopathological factors**		**Overall survival!!break (*****n*** **=** **364)**			**Progression-free survival!!break (*****n*** **=** **366)**		
		***N***	**Hazard ratio**	***P* value**	***N***	**Hazard ratio**	***P* value**
Sex	Female	118	0.26 (0.16–0.4)	**3.2E−10**	120	0.37 (0.21–0.65)	**4.3E−04**
	Male	246	0.31 (0.16–0.59)	**1.8E−04**	246	0.36 (0.24–0.52)	**3.8E−08**
Stage	1	170	0.33 (0.17–0.62)	**3.3E−04**	170	0.41 (0.23–0.75)	**2.6E−03**
	2	83	0.4 (0.18–0.9)	**0.022**	84	0.32 (0.17–0.59)	**1.6E−04**
	3	83	0.37 (0.2–0.67)	**6.7E−04**	83	0.57 (0.31–1.05)	0.066
	4	4			5		
Grade	1	55	0.34 (0.13–0.94)	**0.031**	55	0.54 (0.24–1.2)	0.12
	2	174	0.26 (0.15–0.45)	**2.4E−07**	175	0.29 (0.18–0.46)	**3.9E−08**
	3	118	0.34 (0.19–0.63)	**3.4E−04**	119	0.41 (0.25–0.68)	**3.7E−04**
	4	12			12		
AJCC T	1	180	0.33 (0.18–0.6)	**1.6E−04**	180	0.42 (0.23–0.75)	**2.5E−03**
	2	90	0.38 (0.18–0.81)	**8.8E−03**	92	0.36 (0.2–0.64)	**3.0E−04**
	3	78	0.35 (0.19–0.64)	**4.2E−04**	78	0.62 (0.34–0.12)	0.12
	4	13			13		
Vascular invasion	Yes	90	0.38 (0.17–0.82)	**0.01**	91	0.51 (0.28–0.92)	**0.023**
	None	203	0.29 (0.17–0.5)	**2.8E−06**	204	0.55 (0.35–0.86)	**8.2E−03**
Race	White	181	0.38 (0.24–0.61)	**3.5E−05**	183	0.42 (0.28–0.64)	**2.2E−05**
	Asian	155	0.16 (0.08–0.3)	**9.2E−11**	155	0.27 (0.17–0.44)	**2.7E−08**
Alcohol consumption	Yes	115	0.39 (0.21–0.75)	**3.5E−03**	115	0.35 (0.21–0.58)	**3E−05**
	None	202	0.31 (0.19–0.5)	**3.6E−07**	204	0.4 (0.26–0.63)	**2.9E−05**
Virus hepatitis	Yes	150	0.33 (0.17–0.63)	**4.1E−04**	152	0.35 (0.21–0.57)	**9.6E−06**
	None	167	0.28 (0.17–0.48)	**6E−07**	167	0.34 (0.21–0.55)	**2.7E−06**

*Bold values indicate P < 0.05*.

### Correlation Analysis Between CLEC3B Expression Levels and Degree of Immune Cell Infiltration in HCC

**Figure 3:**
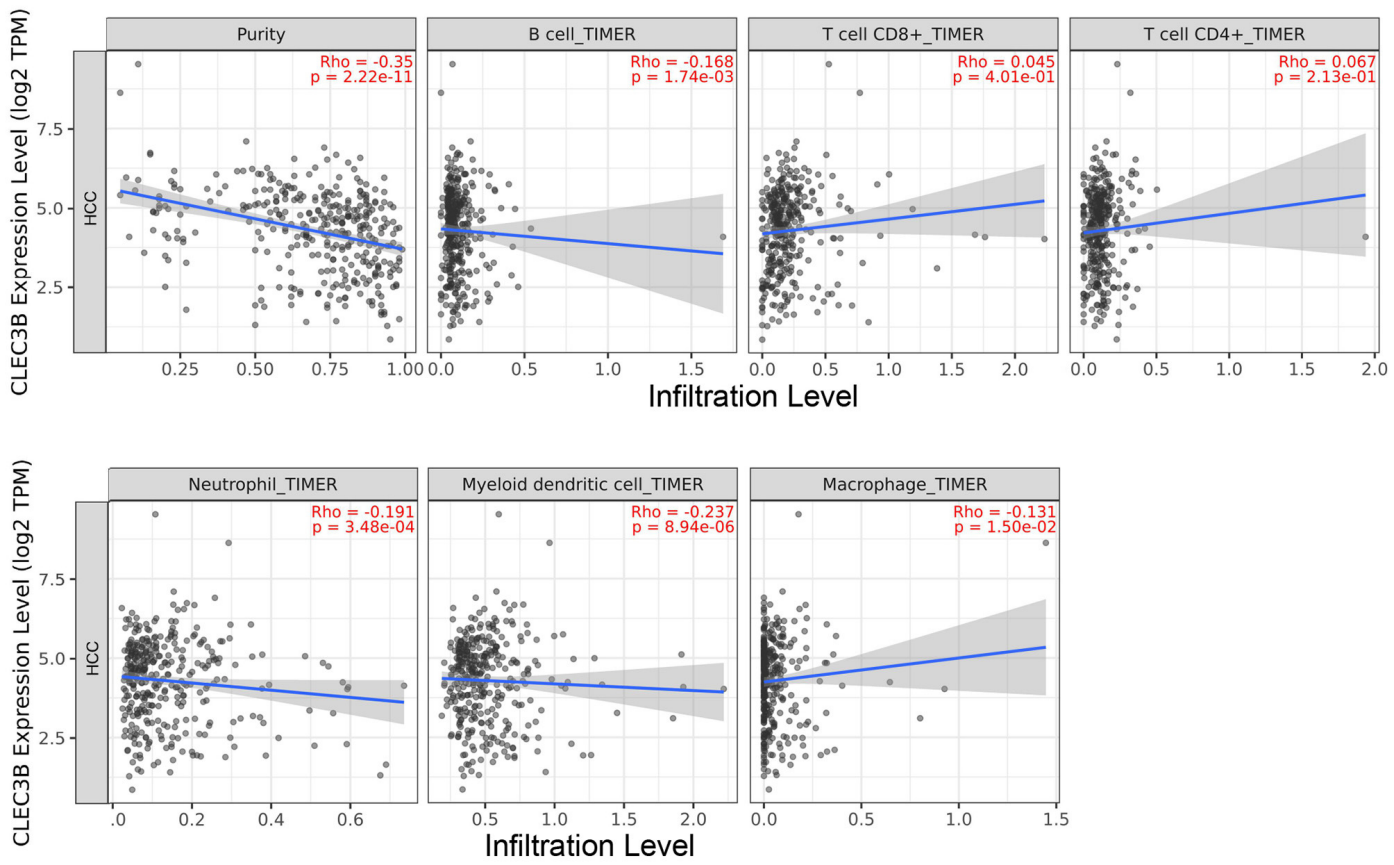
Correlation analysis between *CLEC3B* expression and immune cell infiltration levels in HCC via TIMER analysis (*n* = 371).

We used TIMER 2.0 to investigate the correlation between the expression of *CLEC3B* and infiltration levels of immune cells in 31 tumor tissues. The expression of *CLEC3B* showed a notable correlation with the purity of the tumor in 20 tumor types. Additionally, a negative correlation with *CLEC3B* expression was observed for B cells in five tumors, CD8+ T cells in four tumors, CD4+ T cells in two tumors, macrophages in two tumors, neutrophils in eight tumors, and dendritic cells in five tumors (Supplementary Figure 3). From a macro point of view, a negative correlation was observed between *CLEC3B* transcriptional levels and the infiltration of B cells (Rho = −0.168, *P* = 1.74e−03), macrophages (Rho = −0.131, *P* = 1.50e−02), neutrophils (Rho = −0.191, *P* = 3.48e−04), and dendritic cells (Rho = −0.237, *P* = 8.94e−06) (Figure 3).

### Relationship Analysis Between CLEC3B mRNA Expression Levels and Biomarkers of Different Immune Cell Subsets

After confirming the negative correlation of *CLEC3B* expression with the infiltration of B cells, macrophages, neutrophils, and dendritic cells, we further investigated the expression levels of biomarker genes in these immune cells or their subsets in HCC tissues to provide a basis for investigating CLEC3B-driven tumorigenesis mechanisms and screening suitable therapeutic targets in the future. By TIMER analysis, we found that *CLEC3B* expression was negatively correlated with *CD68* (TAM biomarkers, *r* = −0.285, *P* = 7.73e−08), *IL-10* (TAM biomarkers, *r* = −0.259, *P* = 1.03e−06), *IRF5* (M1 macrophage biomarker, *r* = −0.141, *P* = 8.70e−03), *ITGAM* (neutrophil biomarker, *r* = −0.273, *P* = 2.67e−07), and *ITGAX* (dendritic cell biomarker, *r* = −0.271, *P* = 3.24e−07). Additionally, a positive correlation was observed between *CCL2* (TAM biomarker, *r* = 0.162, *P* = 2.60e−03), *CD1c* (dendritic cell biomarker, *r* = 0.214, *P* = 6.39e−05), and *CCR7* (neutrophil biomarker, *r* = 0.157, *P* = 3.47e−03) (Table 2).

**Table 2:** Correlation analysis between *CLEC3B* and related genes and markers of immune cells via TIMER.

**Description**	**Gene markers**	**Purity**	
		**Correlation**	***P* value**
B cell	CD19	−0.072	0.183
	CD79A	0.011	0.843
Tumor-associated macrophage	CCL2	0.162	**2.6E−03**
	CD68	−0.285	**7.73E−08**
	IL10	−0.259	**1.03E−06**
M1 Macrophage	IRF5	−0.141	**8.7E−03**
	PTGS2	0.003	0.962
M2 Macrophage	CD163	−0.013	0.809
	VSIG4	−0.043	0.424
	MS4A4A	−0.049	0.363
Neutrophils	CEACAM8	−0.094	0.0804
	ITGAM	−0.273	**2.67E−07**
	CCR7	0.157	**3.47E−03**
Dendritic cell	HLA-DPB1	0.015	0.783
	HLA-DQB1	−0.056	0.303
	HLA-DRA	−0.055	0.309
	HLA-DPA1	−0.017	0.76
	CD1c	0.214	**6.39E−05**
	NRP1	−0.062	0.25
	ITGAX	−0.271	**3.24E−07**

*Bold values indicate P < 0.05*.

## Discussion

HCC, which has a high mortality rate, ranks second to lung cancer in malignancy (Bray et al., 2018). Only 20% of patients with HCC are treated in a timely manner owing to early diagnosis; however, the rest are not eligible for cancer-specific treatment due to late-stage diagnosis (Huang et al., 2010). In this study, using a bioinformatics analysis method with public resource databases, we revealed that *CLEC3B* expression was decreased in HCC and was correlated with a poor survival rate in patients with HCC. Additionally, the expression of *CLEC3B* was negatively correlated with differential immune cell infiltration and various immune biomarkers. These results indicate a potential mechanism by which the expression of *CLEC3B* might adjust tumor immunity by modulating the infiltration of HCC immune cells. Our study demonstrated that *CLEC3B* could be a potential prognostic biomarker and might be involved in tumor immune cell infiltration in HCC.

Owing to miRNA-mediated post-transcriptional regulation, mRNA expression patterns do not necessarily indicate the final protein expression level, especially in tumorigenesis. In this study, however, mRNA was used as the monitoring biomarker in the early stage of cancer or prognosis due to identifiable interaction processes at the gene level. Furthermore, mRNA detection is more rapid and convenient than that of proteins. Analysis of *CLEC3B* mRNA expression levels in normal and tumor tissues indicated that the expression of *CLEC3B* was notably downregulated in most tumors, with identical expression patterns in HCC in the different databases used. Liu et al. (2018) found that *CLEC3B* functions in the mitogen-activated protein kinase pathway and is positively correlated with proliferation inhibitors. Thus, the downregulation of *CLEC3B* expression in tumor tissue contributes to carcinogenesis by uncontrolled proliferation of tumor cells (Liu et al., 2018). Sun et al. (2020) indicated that CLEC3B might function as a tumor suppressor in tumor–immune interactions (Sun et al., 2020). Other researchers found that downregulated *CLEC3B* promoted the migration, invasion, and epithelial–mesenchymal transition of tumor cells (Dai et al., 2019). Although the function of CLEC3B in tumorigenesis is still controversial, a large number of studies have shown that CLEC3B plays a role in immune infiltration and immune activation, and low *CLEC3B* expression is associated with poor prognosis in various tumors, which is consistent with our results of HCC, thus highlighting the importance of monitoring the expression level of *CLEC3B* to prevent postoperative recurrence.

We investigated the association between the expression levels of *CLEC3B* and four important survival parameters by Kaplan–Meier plotter with more than 300 samples of HCC patients. Here, we found that high transcription levels of *CLEC3B* were correlated with the overall survival of HCC patients as well as disease-specific survival, suggesting that high levels of *CLEC3B* mRNA are beneficial to the survival of HCC patients and may improve the resistance of patients to other diseases to a certain extent. In addition, by analyzing progression-free survival, we can conclude that high levels of *CLEC3B* expression contribute to the inhibition of HCC deterioration. A similar conclusion was also found in the investigation of relapse-free survival, indicating that *CLEC3B* expression was positively correlated with prognosis of HCC patients treated with multiple treatment methods. Therefore, it is plausible to consider *CLEC3B* as a potential prognostic biomarker in HCC.

To further investigate the mechanism of CLEC3B in HCC tumorigenesis, we analyzed the correlation between *CLEC3B* gene expression and immune cell infiltration. Dendritic cells are special antigen-presenting cells that play a major role in activating T lymphocytes with anti-tumor effects (Zhang et al., 2019; Wculek et al., 2020). In contrast, high expression of TAMs has been shown to be associated with lymphatic metastasis, tumor burden, and poor prognostic survival (Kim, 2013). Our results revealed high activity of TAM infiltration in HCC, indicating that low expression of *CLEC3B* could promote HCC tumorigenesis by increasing TAMs. This may explain the relationship between low *CLEC3B* expression or high levels of immune cell infiltration and the low survival rate of patients with HCC. This finding could contribute to the development of new immunotherapeutic agents for patients with HCC who do not respond to existing immunosuppressive checkpoint inhibitors. However, TAMs have been reported to be able to synergize with T cells to exert anti-tumor effects under specific stimuli (Kim, 2013), suggesting that the timing of operation should be considered. The association between the infiltration activity of immune cells and *CLEC3B* expression in HCC further suggests that CLEC3B may be involved in the oncogenesis and progression of HCC, thus demonstrating the potential of *CLEC3B* as a biomarker gene.

In recent years, tumor immunotherapy has developed rapidly with a growing recognition of the role of the immune system in the occurrence and development of cancer. The tumor microenvironment is an active research area to search for related genes that can serve as diagnostic and prognostic markers or therapeutic targets (Altorki et al., 2019). In our study, the expression levels of some biomarker genes in immune cells were negatively correlated with *CLEC3B* expression, which was consistent with the negative correlation between *CLEC3B* expression and immune cell infiltration. However, some biomarker genes were positively correlated. This inconsistency suggests the complexity of tumorigenesis and immunization. Nevertheless, our results are still relevant and provide a preliminary direction for selecting therapy targets. The tumor microenvironment comprises various immunocyte subtype cells, such as M1 macrophages and M2 macrophages (Ino et al., 2013). Different cell subtypes play different roles in the immune microenvironment. M1 mcrophages exert anti-tumor effects following activation by Th1 cytokines, whereas M2 macrophages show pro-tumor activity (den Breems and Eftimie, 2016). We observed a significant correlation between *CLEC3B* and *IRF5* (biomarker of M1 macrophages), and speculated that *IRF5* might be a novel potential target gene for HCC treatment. Additionally, biomarker genes (*ITGAM* and *ITGAX*) related to the expression of *CLEC3B* in neutrophils and dendritic cells could serve as new research targets in the future. It should be noted that our results were based on the analysis of big data from various databases, and we collected the data as comprehensively as possible. However, this finding can only provide a preliminary theoretical basis and needs to be further verified by follow-up studies and clinical trials.

## Data Availability Statement

The datasets presented in this study can be found in online repositories. The names of the repository/repositories and accession number(s) can be found in the article/Supplementary Material.

## Author Contributions

X-WX and XL contributed to the concept and wrote the manuscript. X-WX and S-SJ designed experiments, performed experiments, and analyzed data. X-WX and S-SJ contributed equally to this study. All authors contributed to the article and approved the submitted version.

## Conflict of Interest

The authors declare that the research was conducted in the absence of any commercial or financial relationships that could be construed as a potential conflict of interest.
