# Supplementary material for: CLEC3B as a Potential Prognostic Biomarker in Hepatocellular Carcinoma
Source: Front Mol Biosci. 2021 Jan 20;7:614034. doi: 10.3389/fmolb.2020.614034 (PMC7855974; doi:10.3389/fmolb.2020.614034)
Supplement: Supplementary file 1 [file Data_Sheet_1.PDF]

SUPPLEMENTARY FIGURE 1

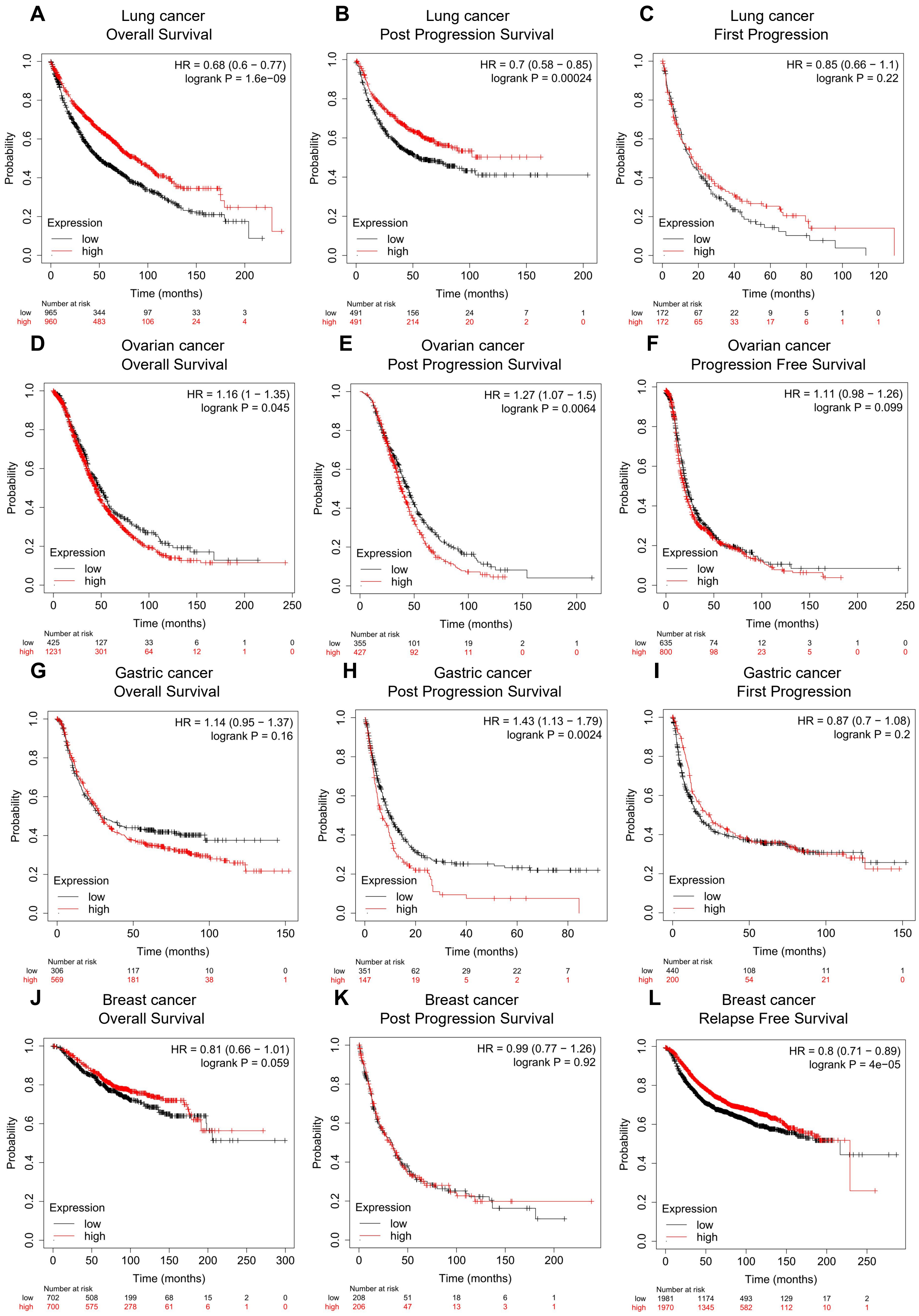

Supplementary Fig. 1. Correlation analysis between *CLEC3B* expression and prognostic values in diverse types of cancers via Kaplan-Meier plotter analysis. (A–C)

The survival curve of overall survival, post progression survival and first progression in lung cancer (n =1925, n =344, n=982). (D–F) The survival curve of overall survival, post progression survival and progression free survival in ovarian cancer (n =1656, n=782, n =1435). (G-I) The survival curve of overall survival, post progression survival and first progression in gastric cancer (n =875, n=498, n =640). (J-L) The survival curve of overall survival, post progression survival and relapse free survival in the breast cancer (n=1,402, n=414, n=3,951).

SUPPLEMENTARY FIGURE 2

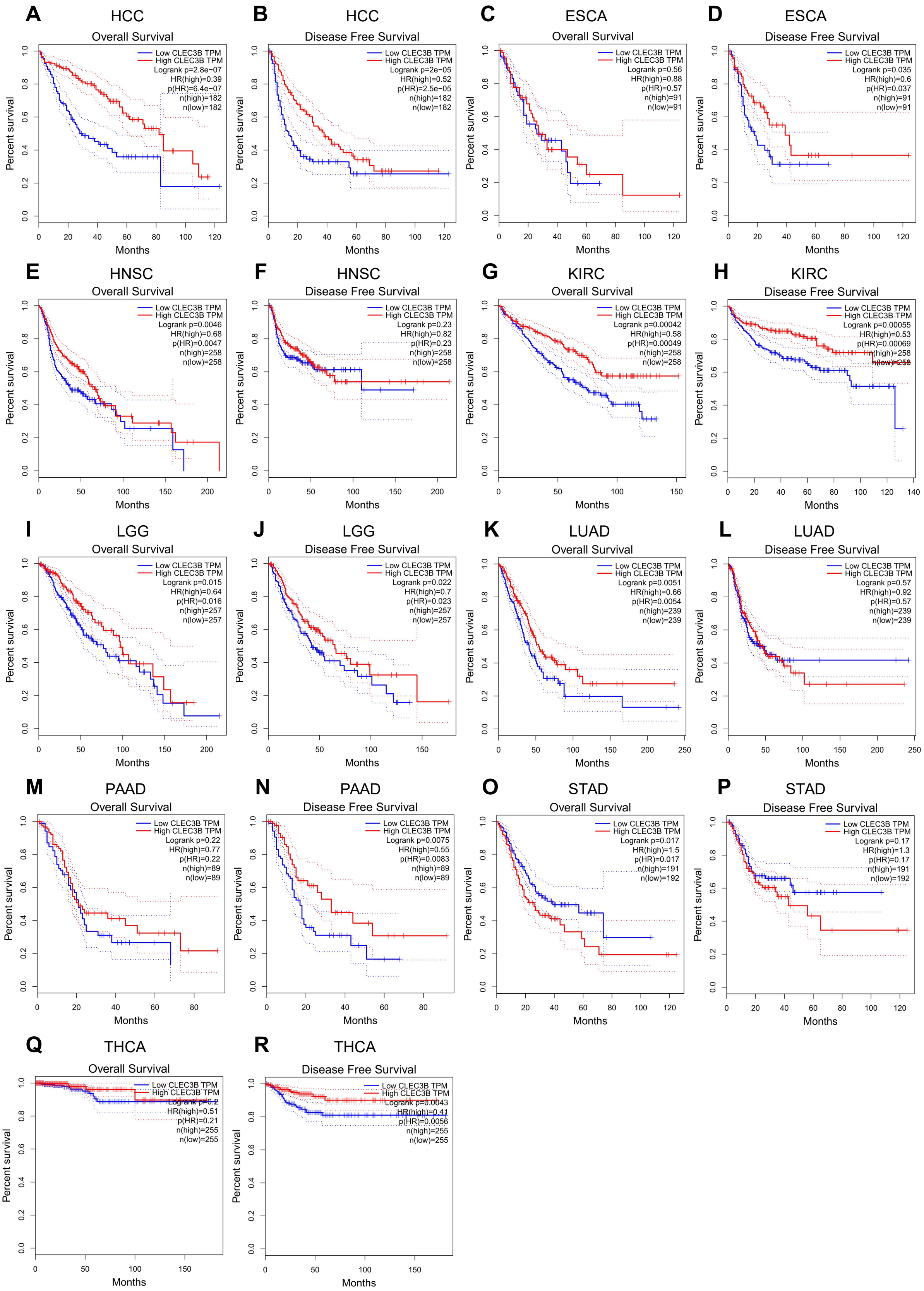

Supplementary Fig. 2. Correlation analysis between *CLEC3B* expression and prognostic values in diverse types of cancers with GEPIA databases. The effects of *CLEC3B* expression on overall survival and disease free survival in Liver

hepatocellular carcinoma (HCC) (A–B), esophageal carcinoma (ESCA) (C–D), head and neck squamous cell carcinoma (HNSC) (E–F), kidney renal clear cell carcinoma (KIRC) (G–H), brain lower grade glioma (LGG) (I–J), lung adenocarcinoma (LUAD) (K–L), pancreatic adenocarcinoma (PAAD) (M–N), stomach adenocarcinoma (STAD) (O–P), thyroid carcinoma (THCA) (Q–R).

SUPPLEMENTARY FIGURE 3

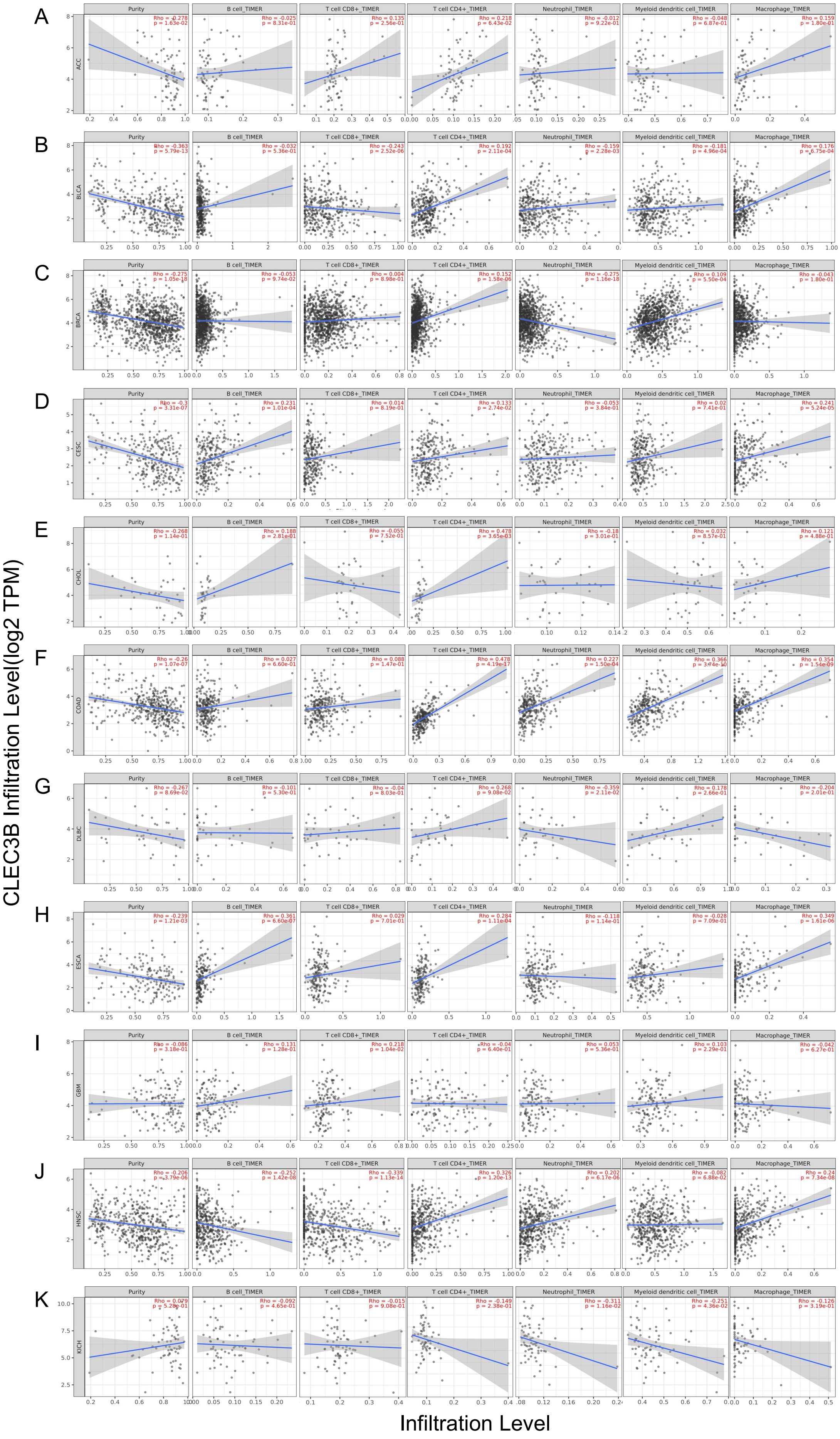

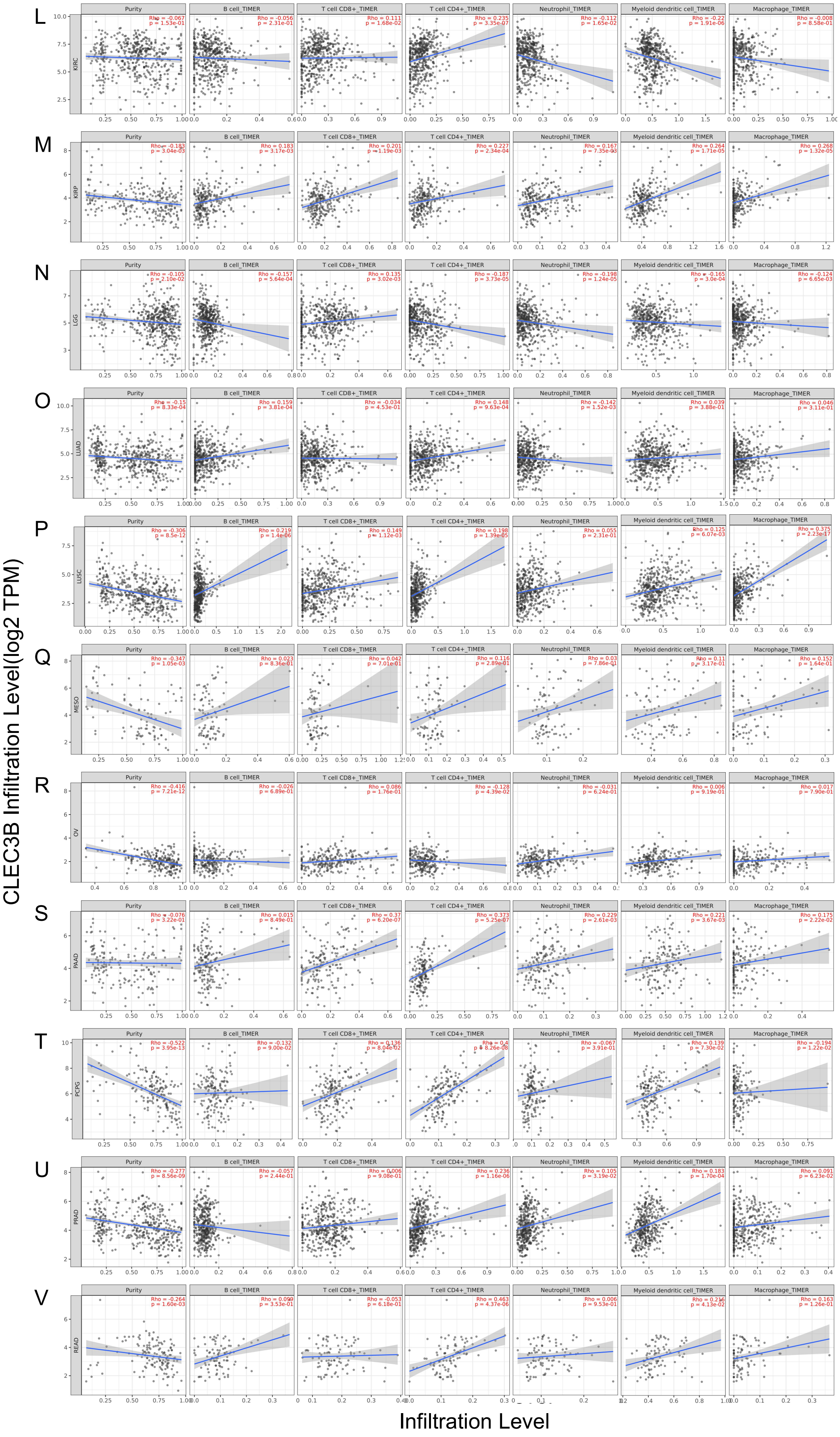

L
M
N
O
P
Q
R
S
T
U
V
Purity
B cell_TIMER
T cell CD8+_TIMER
T cell CD4+_TIMER
Neutrophil_TIMER
Myeloid dendritic cell_TIMER
Macrophage_TIMER
KIRC
KIRP
LGG
LUAD
LUSC
MESO
OV
PAAD
PCPG
PRAD
READ
CLEC3B Infiltration Level(log2 TPM)
Infiltration Level

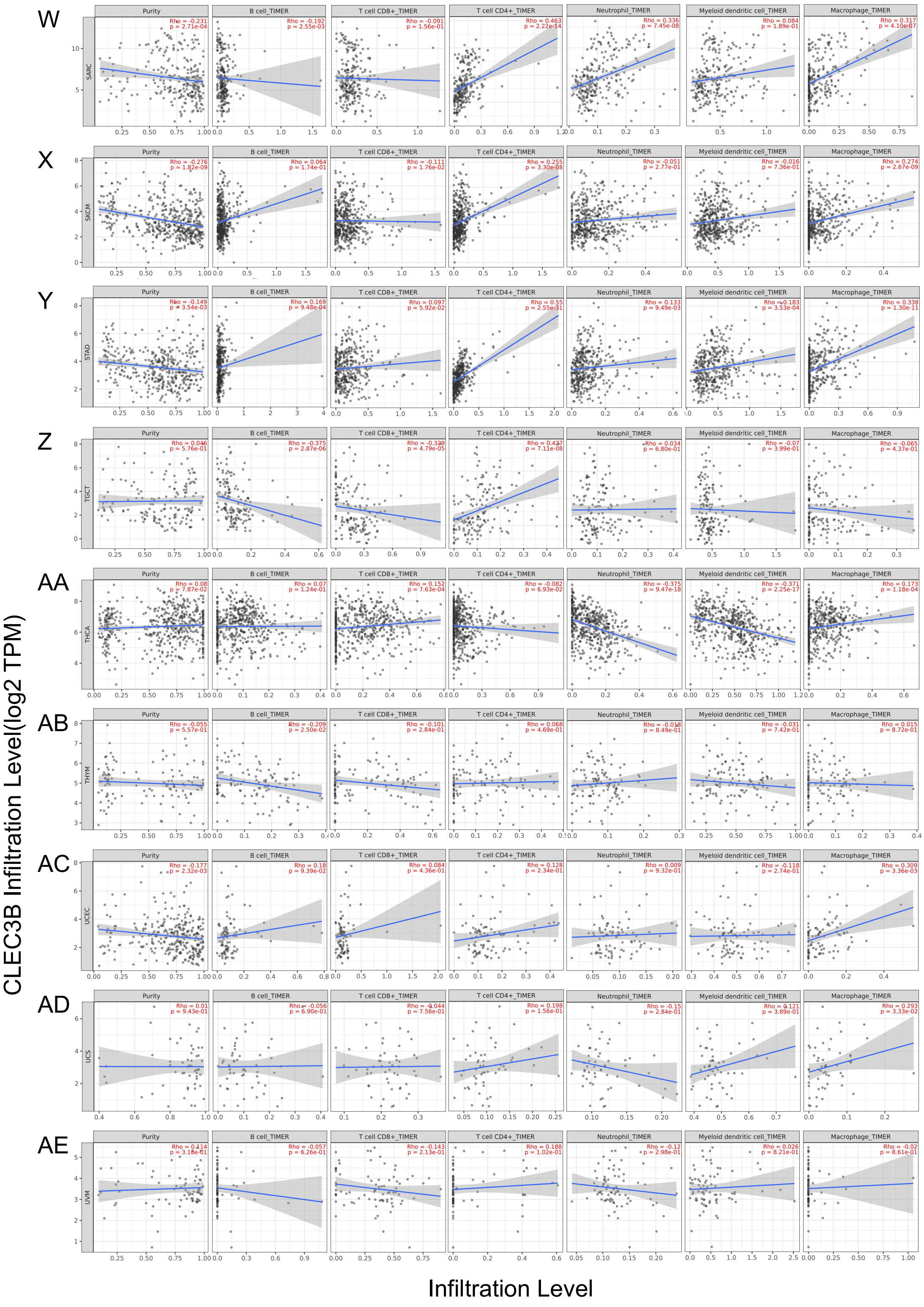

Supplementary Fig. 3. Correlation analysis between *CLEC3B* expression and immune cell infiltration levels in diverse types of cancers via TIMER 2.0 analysis. (A) adrenocortical carcinoma (ACC), (B) bladder urothelial carcinoma (BLCA), (C) breast invasive carcinoma (BRCA), (D) cervical and endocervical adenocarcinoma (CESC), (E) cholangiocarcinoma (CHOL), (F) colon adenocarcinoma (COAD), (G) diffuse large B-cell lymphoma (DLBC), (H) esophageal carcinoma (ESCA), (I)

glioblastoma multiforme (GBM), (J) head and neck squamous cell carcinoma (HNSC), (K) kidney chromophobe (KICH), (L) kidney renal clear cell carcinoma (KIRC), (M) kidney renal papillary cell carcinoma (KIRP), (N) lower grade glioma (LGG), (O) lung adenocarcinoma (LUAD), (P) lung squamous cell carcinoma (LUSC), (Q) mesothelioma (MESO), (R) ovarian serous cystadenocarcinoma (OV), (S) pancreatic adenocarcinoma (PAAD), (T) pheochromocytoma and paraganglioma (PCPG), (U) prostate adenocarcinoma (PRAD), (V) rectum adenocarcinoma (READ), (W) sarcoma (SARC), (X) skin cutaneous melanoma (SKCM), (Y) stomach adenocarcinoma (STAD), (Z) testicular germ cell tumors (TGCT), (AA) thyroid carcinoma (THCA), (AB) thymoma (THYM), (AC) uterine corpus endometrial carcinoma (UCEC), (AD) uterine carcinosarcoma (UCS), (AE) uveal melanoma (UVM).

SUPPLEMENTARY FIGURE 4

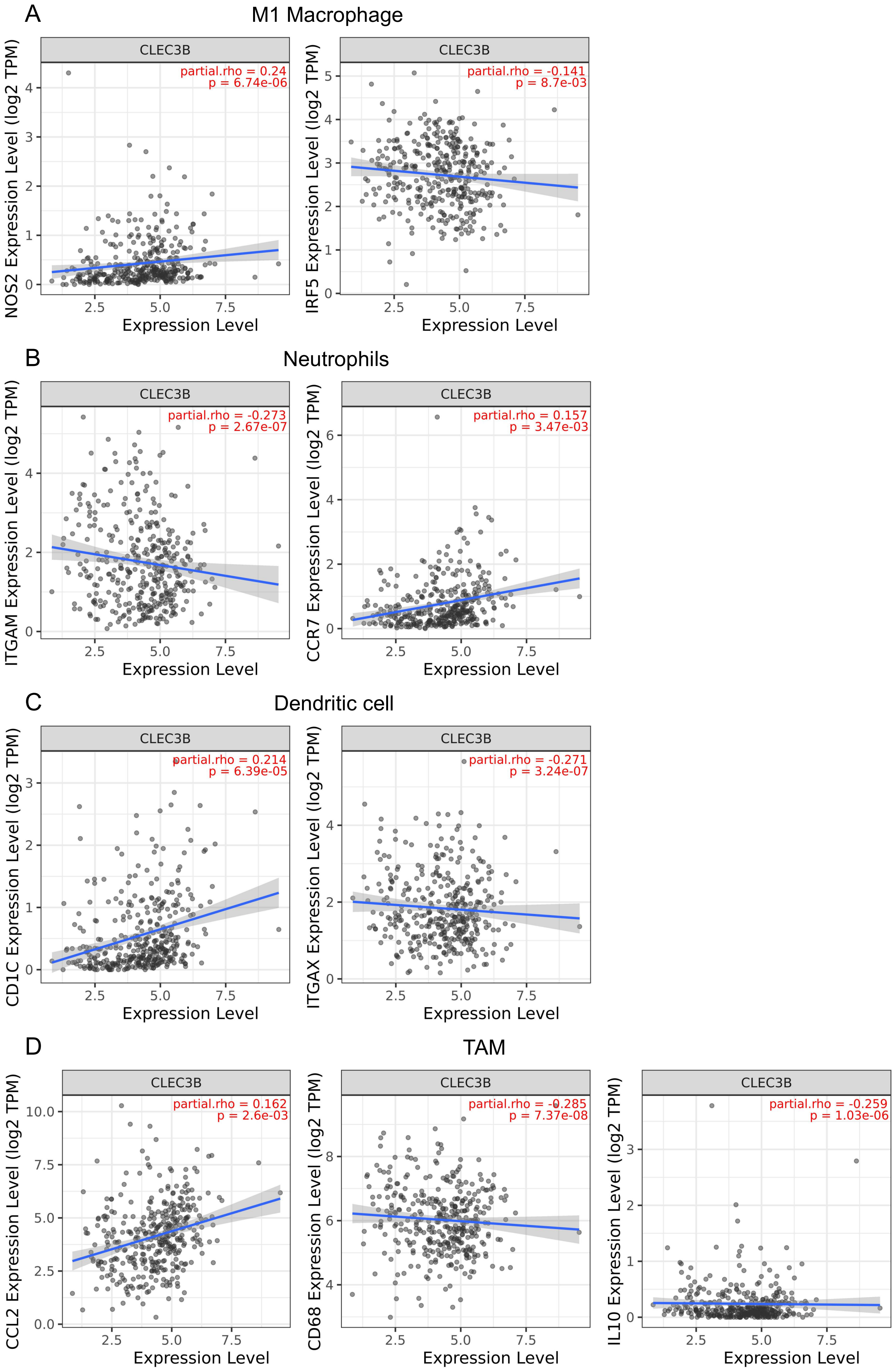

Supplementary Fig. 4. Correlation analysis between *CLEC3B* expression and expression levels of immune cell biomarker genes in patients with HCC via TIMER 2.0 analysis.
